# Lunar Megaregolith Structure Revealed by GRAIL Gravity Data

**DOI:** 10.1029/2021GL095978

**Published:** 2021-11-22

**Authors:** Kristel Izquierdo, Michael M. Sori, Jason M. Soderblom, Brandon C. Johnson, Sean E. Wiggins

**Affiliations:** ^1^ Department of Earth, Atmospheric, and Planetary Sciences Purdue University West Lafayette IN USA; ^2^ Department of Earth, Atmospheric and Planetary Sciences Massachusetts Institute of Technology Cambridge MA USA; ^3^ Department of Physics and Astronomy Purdue University West Lafayette IN USA

**Keywords:** gravity, ejecta, porosity, Moon, Bouguer, megaregolith

## Abstract

We use gravity data from NASA's GRAIL mission to characterize the porosity structure of the upper lunar crust. We analyze the gravitational anomalies produced by the porosity of craters with diameters *D* between 10 and 30 km. We find that the gravitational signature of craters changes significantly at $D=16.4_{-0.6}^{+1.4}$ km, which is related to a discrete change in porosity at a depth ∼3–5 km. We propose that this discrete porosity change reveals the location of the boundary between large‐scale basin ejecta and the deeper less porous portion of the megaregolith, known as the structurally disturbed crust. The ejecta thickness can help constrain models of material transport and mixing on the Moon and, because the ejecta layer acts as an insulating blanket, models of heat flow and magmatism.

## Key Points

- We detect a significant change in the trend between Residual Bouguer Anomaly (RBA) and crater diameter for craters in the range 10–30 km
- The change in RBA trend implies the existence of a porosity boundary at 3–5 km depth, revealing the thickness of large‐scale basin ejecta
- The newly inferred global thickness of the ejecta layer provides constraints on mixing, heat flow, and magmatism models of the Moon

## 1 Introduction

Porosity controls the thermal conductivity and permeability of rocks (Gruescu et al., 2007; Labus & Labus, 2018; Nelson, 1994). On the Moon, an average porosity of 12% extends down to a few kilometers below the surface and substantial porosity extends down to tens of kilometers (Wieczorek et al., 2013). Understanding the evolution and present distribution of this large percentage of porosity is key to understanding the thermal and magmatic evolution of the Moon itself and of planetary bodies in general. Impact cratering is the major process that modifies crustal porosity, and the relationship between the properties of an impact (angle, size, velocity, etc.) and the distribution of the porosity it induces has been modeled before (e.g., Collins, 2014; Johnson et al., 2021; Milbury et al., 2015; Wiggins et al., 2021; Wünnemann et al., 2006).

The lunar megaregolith is the uppermost portion of the lunar crust comprising the most broken‐up layers by impact cratering. It is composed of surficial regolith, large‐scale basin ejecta, crust structurally disturbed by crater collapse and crust fractured in situ. The megaregolith structure is constrained by modeling the lunar cratering evolution with time, photogeologic properties, seismic data, etc (Hörz et al., 1991; Oberbeck et al., 1973; Richardson & Abramov, 2020; Thompson et al., 1979; Wiggins et al., 2019). Using these data, it has been proposed that the large‐scale basin ejecta layer, containing the ejecta from the cumulative cratering of the Moon and dominated by the ejecta of lunar basins, is ∼1.5–2 km thick (Hörz et al., 1991; Richardson & Abramov, 2020; Thompson et al., 1979; Warren et al., 1991). However, gravity data have not been used as a constraint in this thickness even though gravity has been sampled globally on the Moon while seismic data has not. Gravitational constraints on the ejecta thickness would provide a global estimate instead of a local one and improve models of lunar heat flow because the large‐scale ejecta acts as an insulating blanket on the Moon. Models of lateral transport and mixing of material on the Moon (e.g., Huang et al., 2017; Petro & Pieters, 2004) would also benefit from an updated estimate of basin ejecta thickness, helping constrain the original composition of distinct regions on the Moon.

Gravity data provide observational constraints on the impact‐induced porosity because the gravity acceleration of a material depends on its grain density and porosity. Global constraints on impact‐induced porosity of the Moon have been obtained by fitting the gravity signal of lunar craters and modeling the conditions of the impact and target material giving rise to these signals (Ding et al., 2018; Milbury et al., 2015; Soderblom et al., 2015; Wahl et al., 2020; Wiggins et al., 2021). Specifically, Soderblom et al. (2015) fit the Residual Bouguer Anomaly (RBA) of craters, which is the mean Bouguer gravity anomaly inside the crater minus the mean Bouguer gravity anomaly in an area surrounding the crater.

For craters with diameters *D* < 200 km, scatter in RBA data for similar‐sized craters is related to variations in composition of the lunar crust while the trend of RBA data with crater size is related to variations of impact‐induced porosity. RBA decreases as crater size increases within the size range *D* < 200 km. This behavior is consistent with modeling that shows that larger craters tend to have more negative Bouguer anomalies (e.g., Collins, 2014) while composition should not vary with crater size because composition is not predicted to be stratified at shallow depths. The RBA of craters with diameters *D* smaller than 27 km has not been computed before because of the limiting resolution of gravity (Konopliv et al., 2014) and crater data (Head et al., 2010) but it was predicted that porosity in the upper 8 km of the crust, as constrained by these craters, might be independent of crater size (Soderblom et al., 2015). The most likely mechanism to explain this independence would be a state of porosity saturation.

Milbury et al. (2015) showed that the RBA of craters is controlled by the amount of pre‐impact porosity of the material, assuming a uniform porosity with depth. An impact reduces the porosity of the target material if the pre‐impact porosity is high enough because the shock wave compacts the void spaces in the material. This compaction increases the bulk density inside the crater and produces a positive RBA. An impact of the same energy would, instead, increase the porosity of the target if the pre‐impact porosity is low enough. Assuming an exponential profile of porosity with depth, instead of a uniform one, would tend to increase the average pre‐impact porosity at which the RBA become positive (Johnson et al., 2021) but differences in the slope of the RBA data with crater size would still relate to differences in the porosity with crater size.

In this work, we use Gravity Recovery and Interior Laboratory (GRAIL) data (Zuber et al., 2013) to constrain the porosity profile of the upper 8 km of the lunar crust to find regions of saturated porosity or discrete changes in the porosity structure that reveal megaregolith layer boundaries. We build onto the work of Milbury et al. (2015) and Soderblom et al. (2015) and compute the RBA per diameter of lunar craters in the size range of 10 km < *D* < 30 km (Section 2). We discuss the implications of our results for lunar geology, thermal evolution, and magmatic evolution (Section 4).

## 2 Gravity and Crater Data

The gravity field model GRGM1200bRM1 obtained by Goossens et al. (2020) is the latest high‐resolution gravity field model of the Moon. With spherical harmonic degrees and orders 1,200, its spatial resolution is 4.5 km by 4.5 km at the Moon's equator. The degree at which the signal‐to‐noise ratio of the model is one (degree strength) is higher than 600 at all locations of the Moon, with most locations having a degree strength above 800. This model was constructed with GRAIL data, which provided global sampling of the lunar gravity field including its far side. We use the free‐air spherical harmonics coefficients of this model and remove the gravity signal of topography to compute the Bouguer gravity anomalies. We use the LOLA2600p topography model with principal‐axis coordinates (Wieczorek, 2015) constructed with data from the Lunar Orbiter Laser Altimeter (LOLA; Smith et al., 2017) and process the Bouguer correction with the Python module SHTOOLS (Wieczorek & Meschede, 2018), assuming a crustal density value of 2,550 kg/m^3^ (Wieczorek et al., 2013).

We filtered out degrees 1–6 and degrees ≥ 650 of the resulting Bouguer gravity field to remove the effect of the crustal dichotomy and to be conservative with the noise of the data, as indicated by the degree strength. We then applied a cosine taper to degrees 600–650 to avoid ringing. Using degrees up to 600 results in a half‐wavelength resolution of 9 km, which sets the minimum size of craters diameter we can use in computing the RBA.

The global data set of lunar craters provided by Robbins (2019) is complete for craters larger than 2 km in diameter. We compute the RBA of 20,730 craters in this data set with diameters *D* between 10 and 30 km. The RBA is calculated by averaging the Bouguer anomaly inside each crater and subtracting the mean Bouguer anomaly withing a 2*R* radius outside of it. The lower limit of *D* is set to be higher than the minimum diameter allowed by the resolution of our Bouguer gravity data and the upper limit is set so we have an overlap with the RBA obtained by Soderblom et al. (2015), where the range of 27–201 km was used. This overlap is useful to compare our results with those of Soderblom et al. (2015), and to identify differences to ensure that the Bouguer correction and RBA data were calculated correctly.

## 3 Model of RBA

**Figure 1:**
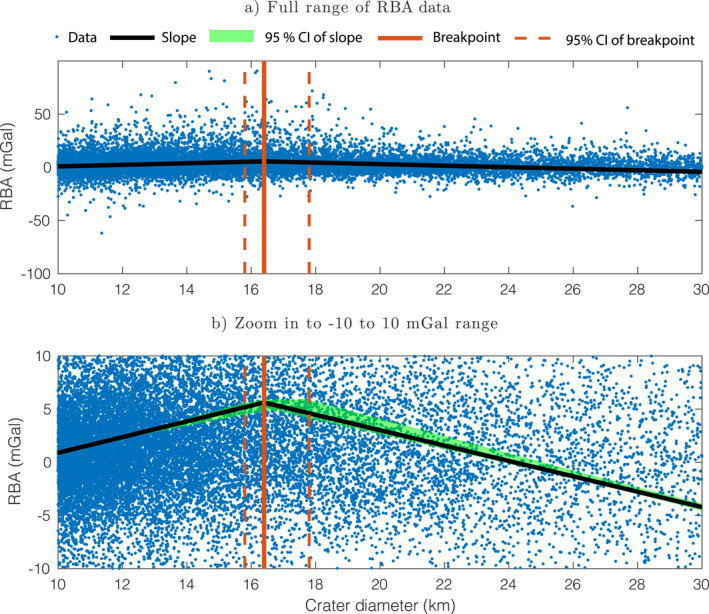
Residual Bouguer anomaly (RBA) of 20,730 lunar craters with diameters between 10 and 30 km and our best fit model. (a) Full range of the RBA data. (b) Zoom in to the −10 to 10 mGal range. The best fit model of the RBA data is a two‐slope model with a positive slope before the breakpoint and a negative slope after it. This breakpoint is located at $D=16.4_{-0.6}^{+1.4}$ km.

Figure 1 shows our calculated RBA values of lunar craters with diameters between 10 and 30 km. The behavior of these data is the same as the one reported in Soderblom et al. (2015) in the overlapping range of diameters 27–30 km, with only minor differences caused by different assumptions in the mean crustal density value and gravity model used. A very important step in this work is to find the model that best represents the obtained RBA data. A flat line at RBA = 0 would point toward porosity saturation while a line with a non‐zero slope would suggest some alternative scenario. We calculated the Bayesian Information Criterion (BIC; Main et al., 1999; Soderblom et al., 2015) of several candidate linear models with one and two slopes to find the model that best describes the RBA data. The BIC is a function of the number of parameters of a model and its likelihood and penalizes models for additional parameters. This allows models with different numbers of free parameters to be compared. For simplicity, we limit candidate models to ones having one or two slopes. Two‐slope models could have two varying slopes or one slope equal to zero (flat).

The BIC value of two‐slope models depends on the location of the breakpoint, or change of slope, so we create two‐slope candidate models with breakpoints varying between 11 <D< 29 km. The model with the highest BIC value of all three groups of models is a two‐slope model with two non‐zero slopes. The Bayes factor ($B_{ab}=e^{BIC_{a}-BIC_{b}}$) between this model and the models with the highest BIC within the two other groups is much higher than 10^2^ which is decisive evidence in favor of this model (Robert et al., 2009). See Section S1 of the Supporting Information S1 for more details.

Figure 1 shows the model that best represents our RBA data. This model is a two‐slope model that changes from a positive slope to a negative slope at $16.4_{-0.6}^{+1.4}$ km. At *D* < 16.4 km, the RBA increases with increasing diameter with a slope of $0.74_{-0.16}^{+0.08}$ mGal/km. At *D* > 16.4 km, the RBA decreases with increasing diameter with a slope of $-0.72_{-0.10}^{+0.04}$ mGal/km. All uncertainties are given as 95% confidence intervals obtained by bootstrapping.

We interpret the change in slope as the result of a boundary between a shallower higher porosity region and a deeper lower‐porosity region because, as Milbury et al. (2015) showed, the relationship between the RBA data and crater diameter increases from negative to positive values with increasing pre‐impact porosity and crater size. Note that the depth to which an impact alters porosity increases with crater diameter. The depth range of the Moon sampled by craters with 10 km <D<16.4 km has then higher porosity than the depth range sampled by craters with 16.4 km <D<30 km. The absolute value of porosity in these regions cannot be constrained, however, because the exact relationship between the RBA and the porosity value has not been described yet.

The relationship between crater diameter and depth range of the porosity induced by the impact is not exact. However, it is well known that the degree of modification of porosity decays with increasing depth from the crater floor. We use the transient crater depth as the maximum depth at which porosity is significantly modified by the impact based on modeling of the porosity induced by impacts (Collins, 2014) and previous RBA studies of porosity (Soderblom et al., 2015) that assume the same depth. Assuming *D*_*t*_ = 4/5*D* where *D*_*t*_ is the transient crater diameter, *D* the final diameter and 1/4*D*_*t*_ < *h*_*t*_ < 1/3*D*_*t*_ where *h*_*t*_ is the transient crater depth (Croft, 1985; Melosh, 1989), we conclude that the boundary between higher and lower pre‐impact porosity is located at a depth of 3–5 km.

**Figure 2:**
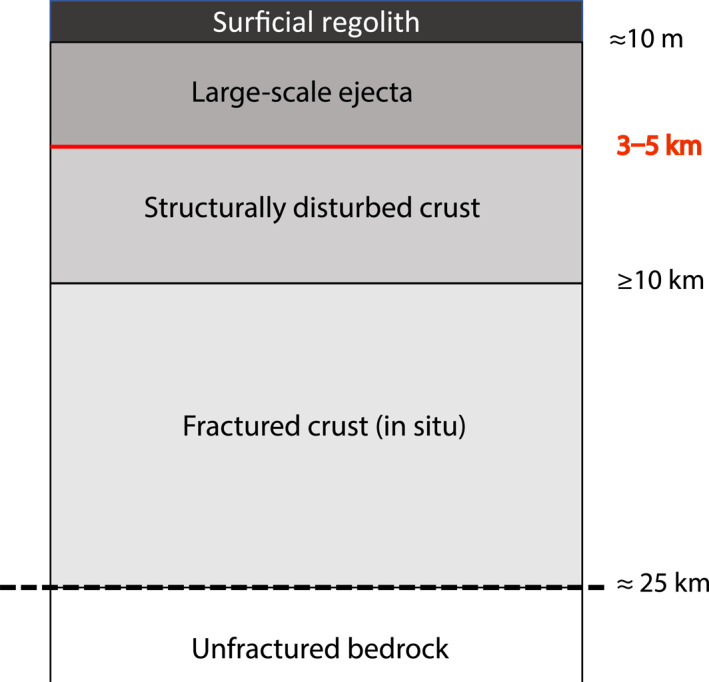
Schematic of the lunar megaregolith modified from Hörz et al. (1991). The location of the boundary between the large‐scale ejecta and the structurally disturbed crust layers, as inferred in this work, is shown in red.

The most likely explanation for a porosity boundary at 3–5 km is that it corresponds to the boundary in the lunar megaregolith between the large‐scale ejecta and structurally disturbed crust. A part of the argument in favor of this interpretation is that fragmentation of the large‐scale ejecta layer is higher than the fragmentation of the structurally disturbed crust. Because fragmentation introduces void space in the material, changes in fragmentation should behave as changes in porosity at large scales. Additionally, the boundary between these two layers had been estimated to be around 1.5–2 km deep (Hörz et al., 1991; Richardson & Abramov, 2020; Thompson et al., 1979; Warren et al., 1991), very close to the 3–5 km depth range we find in this work. Figure 2 shows our interpretation of the lunar megaregolith structure from this work, which is modified from a figure in Hörz et al. (1991).

The RBA data is sensitive to the mean crustal density value used when calculating the Bouguer correction. The inferred depth of 3–5 km we propose is obtained using the best estimation of the mean crustal density which is 2,550 kg/m^3^ (Wieczorek et al., 2013). Varying the mean crustal density value changes the location of the breakpoint of the corresponding best fitting model and the range of the inferred porosity boundary at depth. The depth of this porosity boundary varies from 2.6 to 7.6 km when varying the assumed mean crustal densities between 2,400 and 2,700 kg/m^3^ (S2 Supporting Information S1).

## 4 Discussion

### 4.1 Comparison With Previous Shallow Porosity Profiles

GRAIL data have been used before to study the porosity structure of the Moon above a 27 km depth using different methods than the one described in this work. Wieczorek et al. (2013) and Besserer et al. (2014) and compared the effective density spectrum of constant, linear, and exponential porosity profiles with the corresponding admittance and effective density of the GRAIL data. While these spectral approaches provide models that are consistent with the gravity data, their models do not necessarily match the porosity profiles expected from the cumulative cratering of the upper crust, the mechanism that controls the porosity structure. The porosity structure we provide in this work (Figure 2) is consistent with Bouguer gravity data from GRAIL and with the cratering mechanism that shapes the porosity structure of the shallow crust. Future work will involve analyzing how layered porosity models fit the effective density spectrum of the Moon and the discrepancies with the proposed layered model in this work and previous linear and exponential porosity profiles.

### 4.2 Surface Heat Flow and Magmatism on the Moon

Given the limited measurements of surface heat flow on the Moon (Langseth et al., 1972, 1976), global variations of this parameter are estimated by models of Th abundance and impact ejecta thickness (Hagermann & Tanaka, 2006; Rolf et al., 2017) because these parameters affect the heat production and thermal conductivity in the crust. Using the assumptions in Rolf et al. (2017) regarding ejecta composition and distribution of Th, a global 3 km mean thickness would result in a heat flux higher than 23 mW/m^2^ at the Apollo 15 and 17 landing sites while the proposed values are 21 and 16 mW/m^2^ respectively (Hagermann & Tanaka, 2006). Future heat flux models should take the higher ejecta thickness we propose into account and revisit assumptions on composition and timing of the formation of this insulating layer.

Our study does not provide specific constraints on the total volume of lunar magmatism. Volumes of total magmatism (intrusive and extrusive) however, depend on heat flux (Laneuville et al., 2013). The ejecta material has a low thermal conductivity, therefore, a thicker ejecta layer acts as better insulation and increases the temperature at depth which enhances magmatism. Using our estimated ejecta layer thickness and the assumptions in Rolf et al. (2017), the thickness of nearside basalts would be higher than 3.4 km. It is then necessary to revisit assumptions regarding the ratio of intrusive to extrusive magmatism, models of how the solidus temperature increases in mantle depletion, etc. in order to match the inferred thickness of basalts, which is around 1–2 km (Evans et al., 2016; Gong et al., 2016).

### 4.3 Ejecta Thickness of the Highlands, SPA and Mare Regions

It has been shown that the Bouguer anomalies of impact craters in the SPA and mare regions are statistically different than those in the highlands. These differences point to differences in the porosity profiles of these regions (Bierson et al., 2016). With this in mind, we compute the best fit models for the RBA data of each region and compare it to the global RBA data.

**Figure 3:**
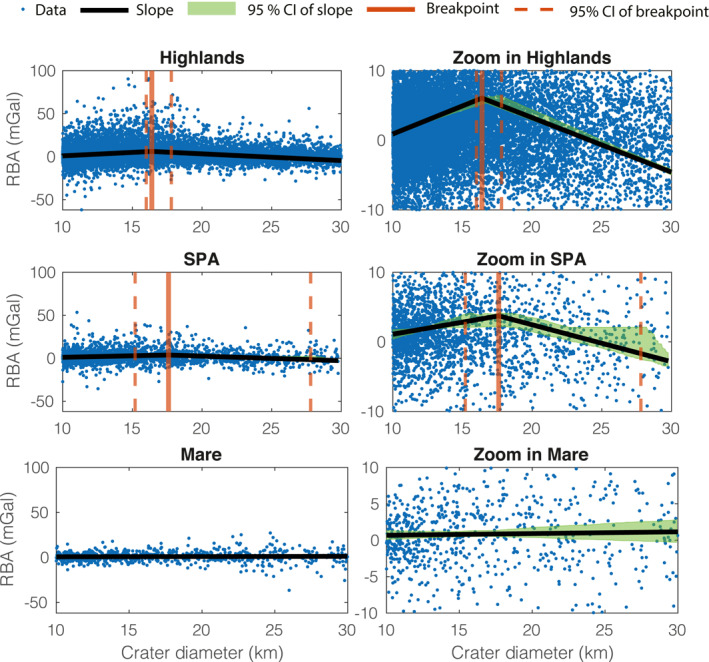
Residual Bouguer Anomaly (RBA) data and best fit models of the Highlands, South Pole‐Aitken (SPA), and Mare regions. The Highlands and SPA regions have two‐slope models with breakpoints at similar locations. Within uncertainty, the inferred vertical porosity profile in these regions is indistinguishable from each other and from one of the global RBA data in Figure 1. The RBA data of the Mare region is best fit by a one‐slope model, likely due to the sparsity of craters in the region. Within uncertainty, the slope could be positive, zero, or negative, making it impossible to infer a porosity profile in this region. See Section 4.3 for details.

Figure 3 shows the RBA of craters in the highlands, SPA, and mare regions and corresponding best fitting models. The highlands and the SPA region are best fit by two‐slope models with two varying slopes: a positive slope before the breakpoint and a negative slope after it. Both highlands and SPA regions are consistent with having the same porosity distribution as the one of the megaregolith described in Section 3: porosity is higher in shallower regions of the crust and it has a discrete change to a lower value in deeper regions. The uncertainty in the location of the breakpoint in the SPA data is much larger, however, due to the fewer number of craters in the SPA compared to the entire Highlands.

The best fit model of the RBA data in the mare regions has no breakpoint. The lack of breakpoint could imply that there is not a discrete change of porosity at depth in this region but it could also be a consequence of the sparsity of the crater data which makes the mare RBA sparser than the highlands or SPA RBA. With fewer data points, more models are consistent with the mare RBA data compare to models for the other regions, which are more uniquely constrained. Because we are using the Bayesian Information Criteria, simpler models are preferred. The slope of the RBA model of the mare region is indistinguishable from a zero slope, a positive or a negative one, making it impossible to say with statistical significance if the porosity in this region is more similar to the shallower or deeper porosity of the highlands region. If the porosity structure of the mare region is in fact different, as was proposed before (Besserer et al., 2014; Bierson et al., 2016), this difference might be due to the emplacement of basalts (low porosity layer) that might make the slope within 10 < *D* < 16 km flatter.

### 4.4 Simple to Complex Crater Transition

The simple to complex crater transition at the Moon has been reported to occur within the range 15 <D< 20 km (Chandnani et al., 2019). This transition diameter represents a possible alternative hypothesis to explain our RBA model breakpoint. However, for the reasons described below, our preferred hypothesis is to attribute the breakpoint to a discrete change in porosity related to a 3–5 km layer of impact ejecta.

The slopes before and after the breakpoint are consistent with a region of high porosity overlying a region of low porosity, which agrees with the expected structure of the upper layers of the lunar megaregolith. The boundary depth is also consistent with independent estimates of the ejecta thickness. Additionally, the mare RBA data does not have a breakpoint even though simple and complex craters exist in the mare region. Lastly, there is no theoretical reason to predict that the simple‐complex crater transition would map to a break in slope in the RBA data. Numerical models that quantify the effects of porosity on resulting crater RBAs at this size range have not been reported.

As previously mentioned, the lack of breakpoint in the mare RBA might be due to the sparsity of crater data in the mare region. This lack of breakpoint alone is then not a robust argument against the simple to complex crater explanation. Considering all arguments in the previous paragraph, however, we support the sensitivity of the RBA slopes to the ejecta layer as an explanation to the break in slope of the global RBA data. Future modeling of the effect of pre‐impact porosity on the RBA of simple and complex craters near the transition diameter, for example expanding the work from Collins (2014) and Milbury et al. (2015), could explicitly test the alternative hypothesis of the simple‐complex transition diameter.

## 5 Conclusion

We investigate the distribution of the porosity in the upper 8 km of the lunar crust by looking at the RBA data of lunar craters in the range 10 km <D<30 km. The RBA points to the existence of a discrete boundary between a shallower, higher porosity layer and a deeper, lower porosity layer. We find this boundary is likely located at a 3–5 km depth. We interpret this geophysical boundary to correspond to the geological boundary between the large‐scale impact ejecta and structurally disturbed crust by crater collapse. We propose that gravity data reveals the structure of the lunar megaregolith. Future work could provide additional evidence of the location of the inferred megaregolith boundary, specifically, by modeling the evolution of the lunar megaregolith in terms of the cumulative effect of cratering on porosity with landscape evolution models (Minton et al., 2015). These results have significant implications regarding the heat flux of the lunar crust and therefore the total volume of magmatism.

## Supporting information

Supporting Information S1

## Data Availability

The GRAIL gravity model GRGM1200bRM1 (Goossens et al., 2020) and LOLA topography model LOLA2600p (Wieczorek, 2015) used in this study are both available for download at https://shtools.github.io/SHTOOLS/python-datasets-constants.html and the Geosciences Node of the NASA Planetary Data System. The Residual Bouguer Anomaly data and plotting codes developed for this work are available at https://zenodo.org/record/5495413.
