# Supplementary material for: Lunar Megaregolith Structure Revealed by GRAIL Gravity Data
Source: Geophys Res Lett. 2021 Nov 22;48(22):e2021GL095978. doi: 10.1029/2021GL095978 (PMC9287069; doi:10.1029/2021GL095978)
Supplement: Supplementary file 1 — Supporting Information S1 [file GRL-48-0-s001.pdf]

Supporting Information for ”Lunar Megaregolith Structure Revealed by GRAIL Gravity Data”

Kristel Izquierdo[1], Michael M. Sori[1], Jason M. Soderblom [2], Brandon C. Johnson [1,3], Sean E. Wiggins[1]

[1]Department of Earth, Atmospheric, and Planetary Sciences, Purdue University, West Lafayette, IN, USA.

[2]Department of Earth, Atmospheric and Planetary Sciences, Massachusetts Institute of Technology, Cambridge, Massachusetts, USA

[3]Department of Physics and Astronomy, Purdue University, West Lafayette, IN, USA.

Contents of this file

1. Text S1 to S4
2. Figures S1 to S8

Introduction

In this supplementary information, we provide further details on 1) the method to find the best fit models of the Residual Bouguer Anomaly (RBA) data, 2) a comparison between the RBA in our study and the one in Soderblom et al. (2015) 3) the sensitivity of the RBA data and best fit models to the assumed value of the mean density of the lunar crust and 4) the sensitivity of the RBA data and best fit models to the truncation degree

of the gravity field and taper used.

Text S1. Models of Residual Bouguer Anomaly.

The Residual Bouguer Anomaly (RBA) of a crater is the average Bouguer anomaly inside the crater minus the average Bouguer anomaly of the surrounding terrain, which is set as the area between $1R$ and $2R$, where R is the radius of the crater. We compute the RBA of all lunar craters with diameters 10 km $< D <$ 30 km and plot the results in Figure 1 of the main text. In this figure, we also show the best fit model of the data which is a two-slopes model with a breakpoint at $D = 16.4^{+1.4}_{-0.6}$ km.

In order to find the best fit model of the RBA data, we employed a Bayesian Information Criterion (BIC) metric (Soderblom et al., 2015; Main et al., 1999) and computed the BIC of several candidate models. Equations 1 and 2 show how the BIC of each model is calculated. L is the likelihood, p is the number of free parameters of the model, ln is the natural log, n is the sample size and ϕ is the sum of the squared residuals between the model and the RBA data.

$$\mathrm{BIC} = \mathrm{L} - \frac{1}{2} p \ln(n) \tag{1}$$

$$\mathrm{L} = -\frac{n}{2} \ln(\phi) \tag{2}$$

We limit the candidate models to three subgroups: one-slope models, two-slope models with one flat slope and two-slope models with two varying slopes. We do not calculate the BIC of models with a higher number of slopes because we are mostly interested in testing

the prediction that the RBA data would be uniform (Soderblom et al., 2015). Having a two-slope model as the model with the highest BIC would disprove that the RBA data is uniform.

Figure S1 shows the BIC of all candidate models. For the two-slopes models, the BIC is a function of where the breakpoint is located (i.e., the diameter at which the change in slope occurs). Breakpoints are located between 12 and 28 km, with intervals of 1 km. The model with the highest BIC is a two-slopes model with two varying slopes. The Bayes factor ($B_{ab} = e^{BIC_a - BIC_b}$) between this model and the models with the highest BIC within the two other groups is much higher than 10^2 which is decisive evidence in favor of this model (Robert et al., 2009). Once knowing that the best fit model is one with two varying slopes, we repeated the calculation of BICs for models with two varying slopes using smaller intervals of breakpoints, increasing the precision of the resulting breakpoint value to $D = 16.4$ km.

We find the uncertainty of the breakpoint and slopes by bootstrapping. At each sample, we remove one data point from the RBA data. After 10 000 samples, we have a distribution of 10 000 values of the breakpoint and 10 000 values for each slope. The 95% confidence interval of breakpoint and slopes are the range of values contained between the 0.025 to 0.975 values of the cumulative probability. The best RBA model with uncertainties is then a two-slope model that changes from a positive slope to a negative one at $16.4^{+1.4}_{-0.6}$ km. At $D < 16.4$ km, the RBA increases with increasing diameter with a slope of $0.74^{+0.08}_{-0.16}$ mGal/km. At $D > 16.4$ km, the RBA decreases with increasing diameter with a slope of $-0.72^{+0.04}_{-0.10}$ mGal/km.

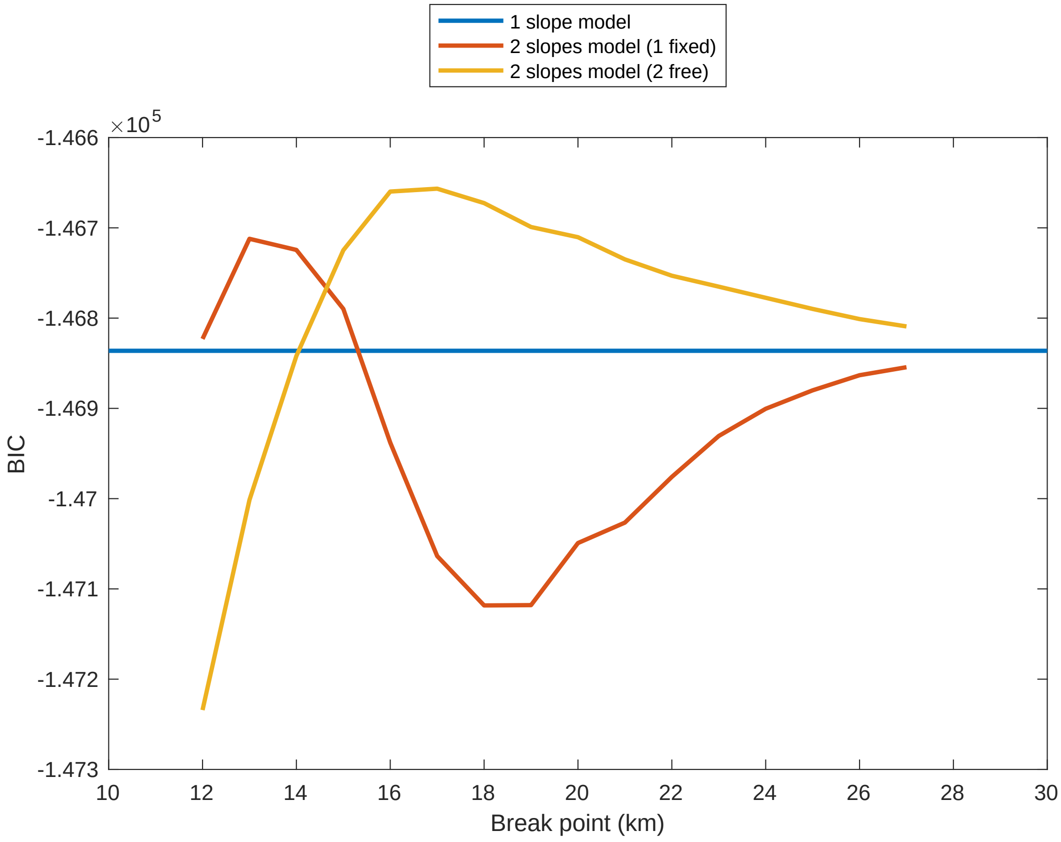

Figure S1. Bayesian Information Criterion (BIC) of candidate models for the Residual Bouguer Anomaly (RBA) data. The RBA data of Figure 1 is best fitted by a two-slopes model with two varying slopes. This result implies that the RBA data is not uniform and there is a boundary of porosity at depth.

Text S2. RBA data comparison.

We compare the RBA data obtained in this study with the one in Soderblom et al. (2015) in order to check if significant differences in the slope around D=30 km exist or if the slopes are consistent with each other. Figure S2 a) shows the complete data sets ranging from D= 10 km to D=188 km. Figure S2 shows a zoom in of both data sets in

the overlapping range $27 < D < 30$ km. Both plots show that, qualitatively, both data sets behave in the same way, without significant vertical shifts between each other.

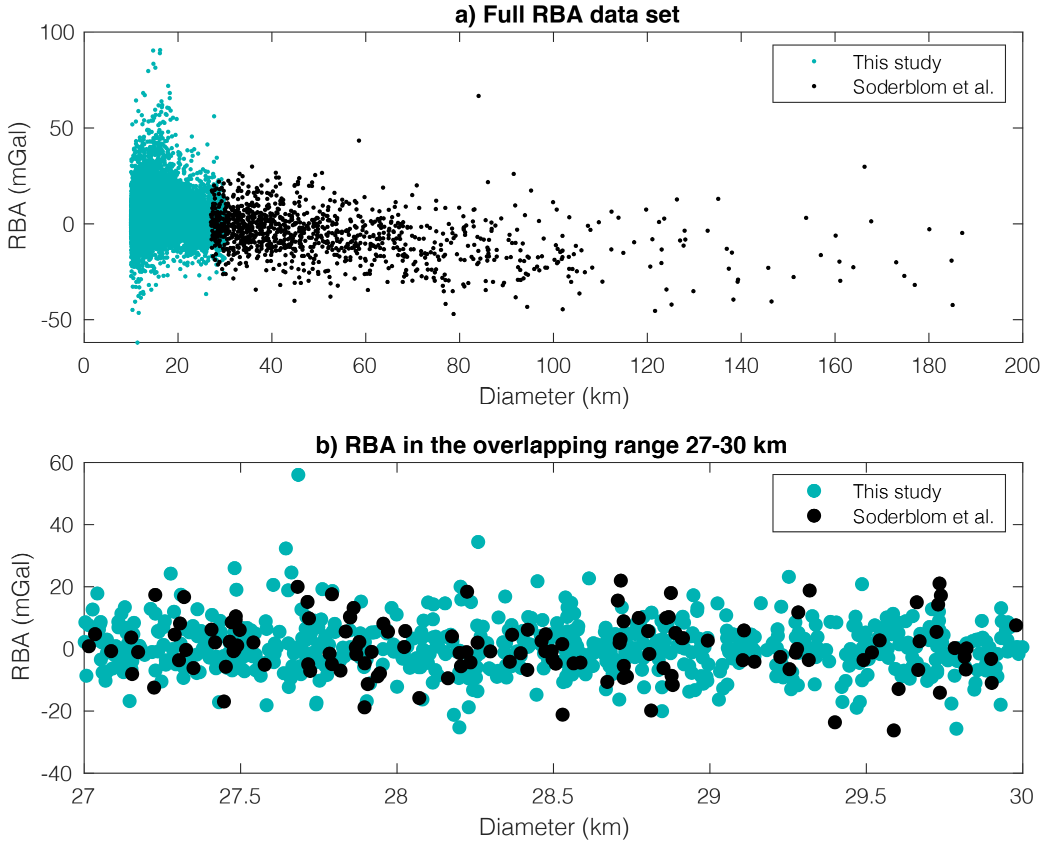

Figure S2. RBA data in the range $10 < D < 200$ km. Black dots show the data from Soderblom et al. (2015) and blue dots show the data for this study. The behavior of both data sets is similar in the overlapping range $27 < D < 30$ km.

We also obtained the best fit model of RBA data within the range of 10 to 93 km (Figure S3) . The best fit model shows that there isn't a significant break in slope around 30 km meaning that the slope before and after 30 km are consistent. Using the merged RBA data, the break in slope is close to our proposed value of 16 km, specifically, it is at $D = 14.8^{+0.4}_{-0.8}$ km. Differences between the break in slope found using the merged data set

and the one found using our dataset alone might be caused by differences in the assumed mean crustal density (2550 vs 2560 kg/m^3), gravity data (Konopliv et al. (2014) versus Goossens et al. (2020)), truncation degrees and crater data (Head et al. (2010) versus Robbins (2019)).

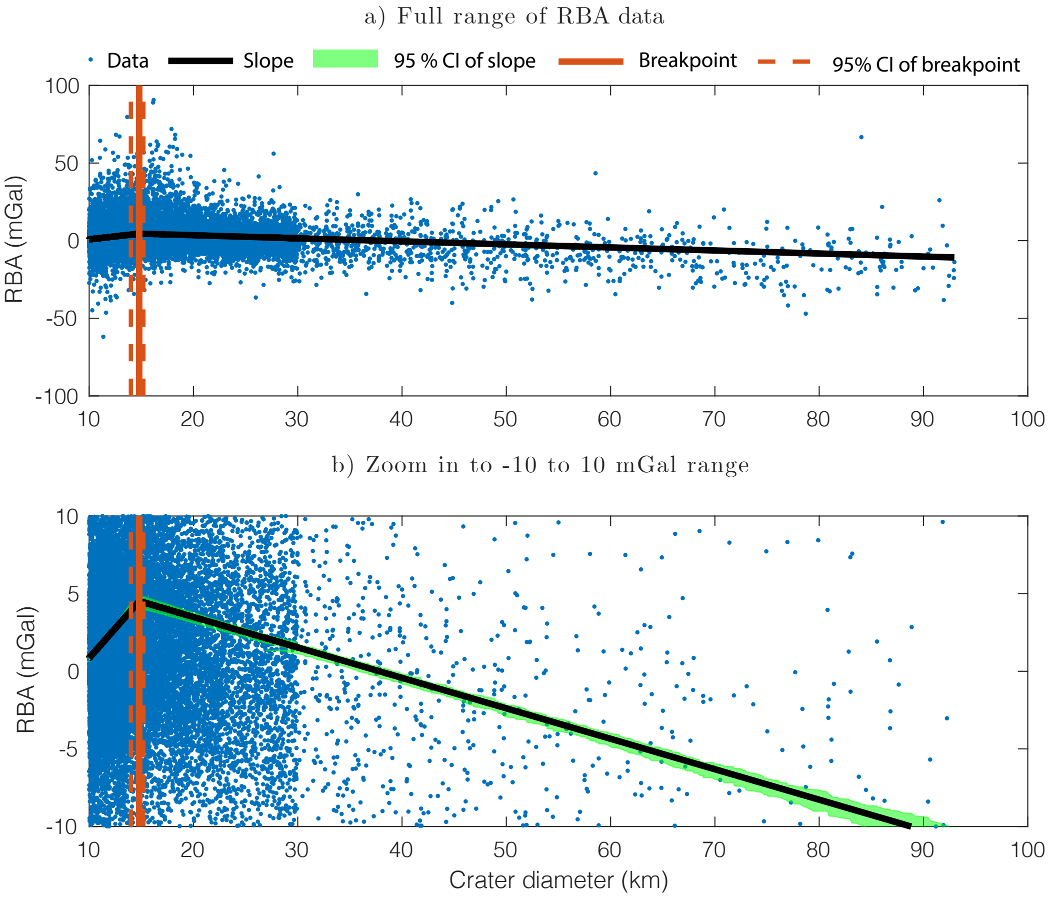

Figure S3. Best fit model of the merged RBA data in the range $10 < D < 200$ km. There is not a break in the slope around $D = 30$ km. The break in slope found is located at $D = 14.8^{+0.4}_{-0.8}$.

We reproduce the RBA obtained in Soderblom et al. (2015) in order to analyze which factors are controlling the differences between data sets. We use the same gravity data,

crater data and truncation degree as in Soderblom et al. (2015) and compute the RBA of craters within the range 27-188 km and corresponding best fit model. We find a breakpoint at $D = 94^{+45}_{-21}$ which is very close to the data reported in Soderblom et al. (2015) of $D = 93^{+47}_{-29}$. The differences between breakpoints are due to the assumed mean crustal values and tapering of the data.

Text S3. RBA for different crustal density assumptions.

The Residual Bouguer Anomaly (RBA) data is sensitive to the value of mean crustal density used in the Bouguer correction. We use the most widely accepted value of 2550 kg/m^3 (Wieczorek et al., 2013) to obtain the global RBA data of lunar craters (Figure 1). As described in the main text, the location of the breakpoint of the best fitting RBA model is $D = 16.4^{+1.4}_{-0.6}$ km and we relate the location D of this breakpoint to a discrete change between a shallower high porosity region and a deeper, lower porosity one, with the boundary located at a depth between 3 and 5 km.

Figure S4 shows the RBA data obtained when using a value of 2400 kg/m^3 for the mean density of the lunar crust, which is a very low value. The breakpoint of the best fitting model is located at $D = 14.0^{+0.4}_{-1.4}$ km. The location of this breakpoint corresponds to a porosity boundary located at a depth between 2.5 and 3.8 km.

Figure S5 shows the RBA data obtained when using a value of 2700 kg/m^3 for the mean density of the lunar crust, which is a very high value. The breakpoint of the corresponding best fitting model is located at $D = 26.4^{+2.2}_{-6.6}$ km. The location of this breakpoint corresponds to a porosity boundary located at a depth between 4.0 and 7.6 km.

Both sets of RBA data have a positive slope for diameters smaller than the breakpoint and a negative slope for diameters larger than the breakpoint. This suggests that, independently of the assumption of mean crustal density, the pre-impact porosity per depth has a discrete change from a region of higher porosity to a region of lower porosity at some depth. The location of this boundary does depend on the assumed mean density, but it would be hard to justify a value closer to 2400 or 2700 kg/m^3 than one closer to 2550 kg/m^3. The best estimate of the mean crustal density then provides the best estimation of the location of the pre-impact porosity boundary which is between 3–5 km.

a) Full range of RBA data

b) Zoom in to -10 to 10 mGal range

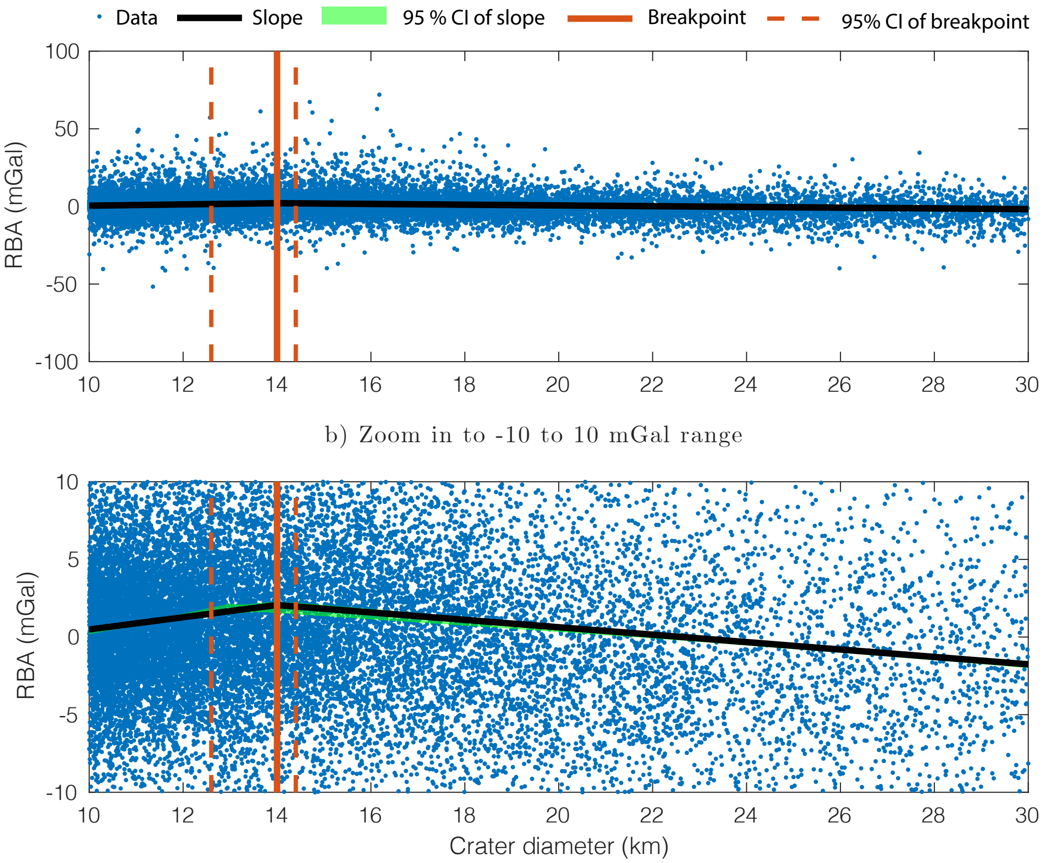

Figure S4. RBA data obtained using a value of 2400 kg/m^3 as the mean density of the lunar crust. The breakpoint is located at $D = 14.0^{+0.4}_{-1.4}$ km.

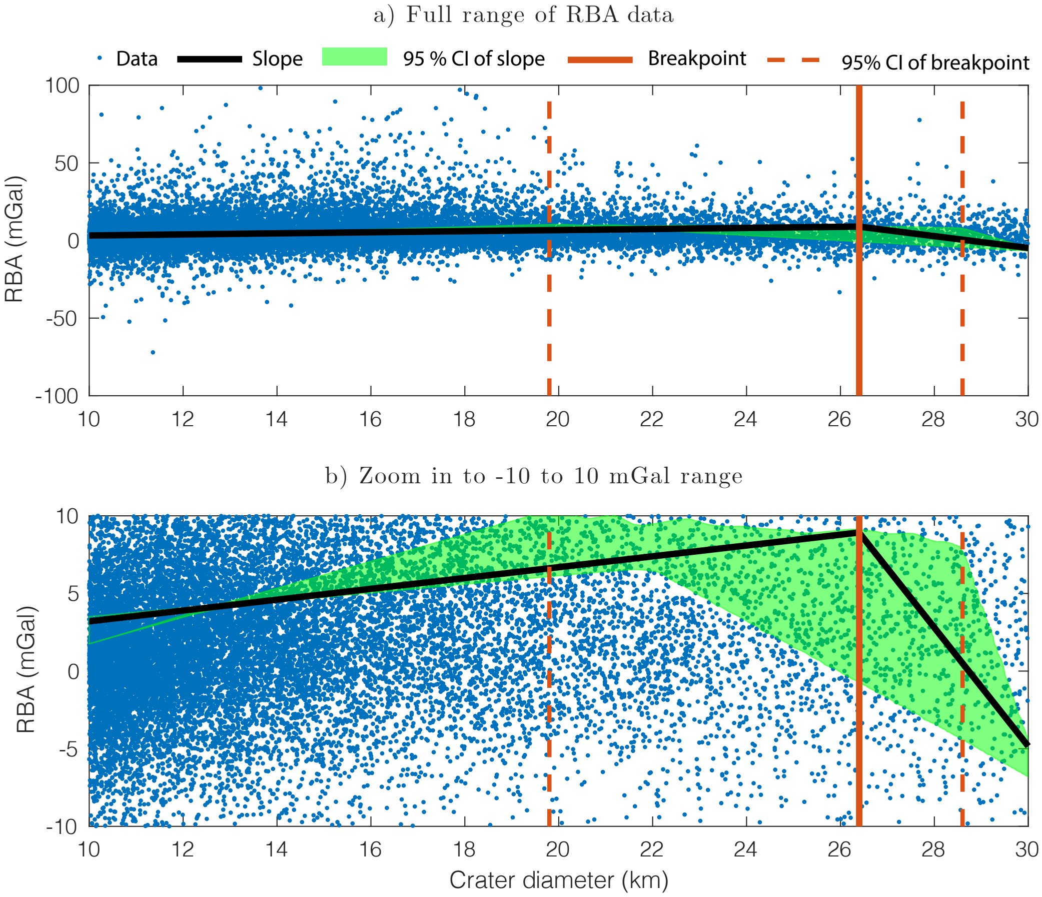

Figure S5. RBA data obtained using a value of 2700 kg/m^3 as the mean density of the lunar crust. The breakpoint is located at $D = 26.4^{+2.2}_{-6.6}$ km.

Text S4. RBA for different truncation degrees and no tapering.

Secondary to the assumption of mean crustal density, the chosen spherical harmonic truncation degree of the gravity model and taper, might affect the resulting RBA data. In this section, we show that the location of the break in slope of RBA data is not affected by the application of a cosine taper. Figure S6 shows the RBA data obtained using a

truncation degree 650 and no cosine taper. The location of the break in slope of the best fit model is $D = 16.4^{+1.2}_{-0.8}$ km showing that only the confidence interval changed, and very slightly, compared to the break in slope obtained using a cosine taper and similar truncation degree, $D = 16.4^{+1.4}_{-0.6}$ (Figure 1 of the main text).

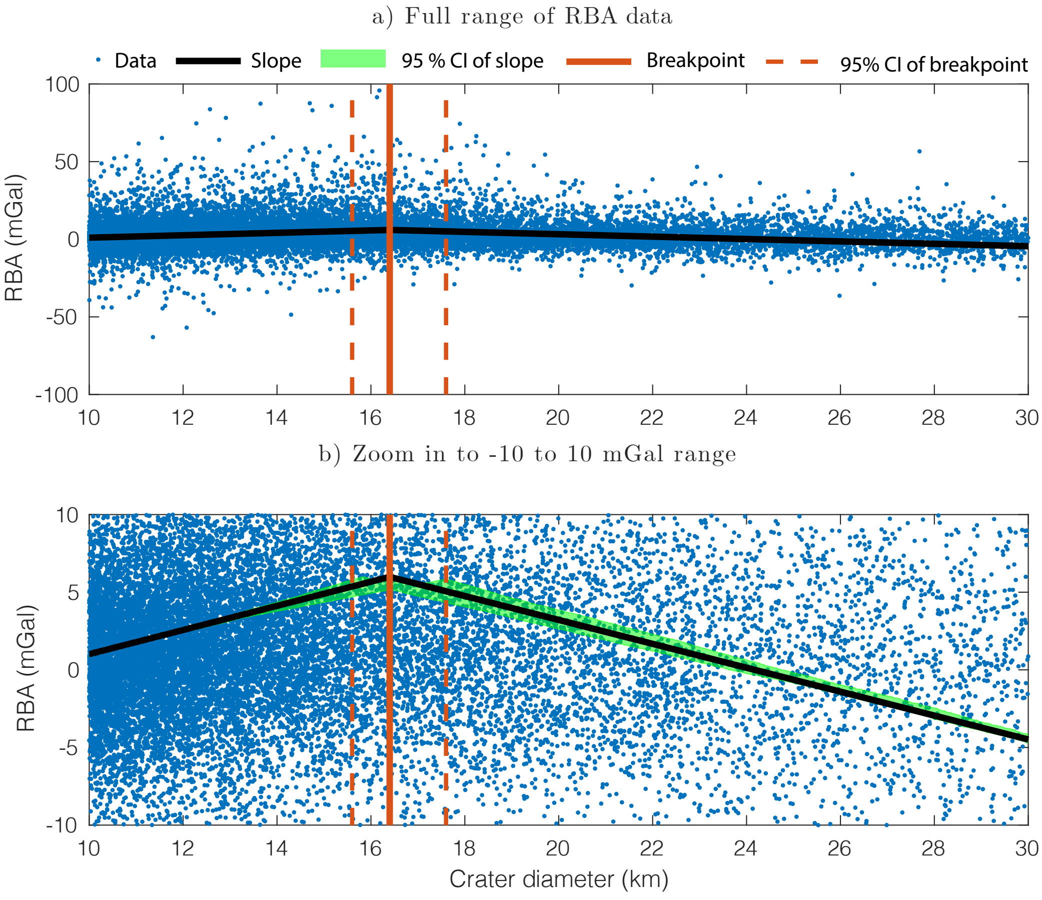

Figure S6. RBA data obtained using a value of 2550 kg/m^3 as the mean density of the lunar crust and truncation degree 650 without tapering. The breakpoint is located at $D = 16.4^{+1.2}_{-0.8}$ km.

We then tested the effect of the chosen truncation degree. Figure S7 shows the RBA data obtained using a truncation degree 640 and no cosine taper. The location of the

break in slope of the best fit model is $D = 17^{+0.6}_{-1.0}$ km showing that there is a small change in the location compared to the one obtained using a truncation degree 650 and no cosine taper, $D = 16.4^{+1.2}_{-0.8}$ km.

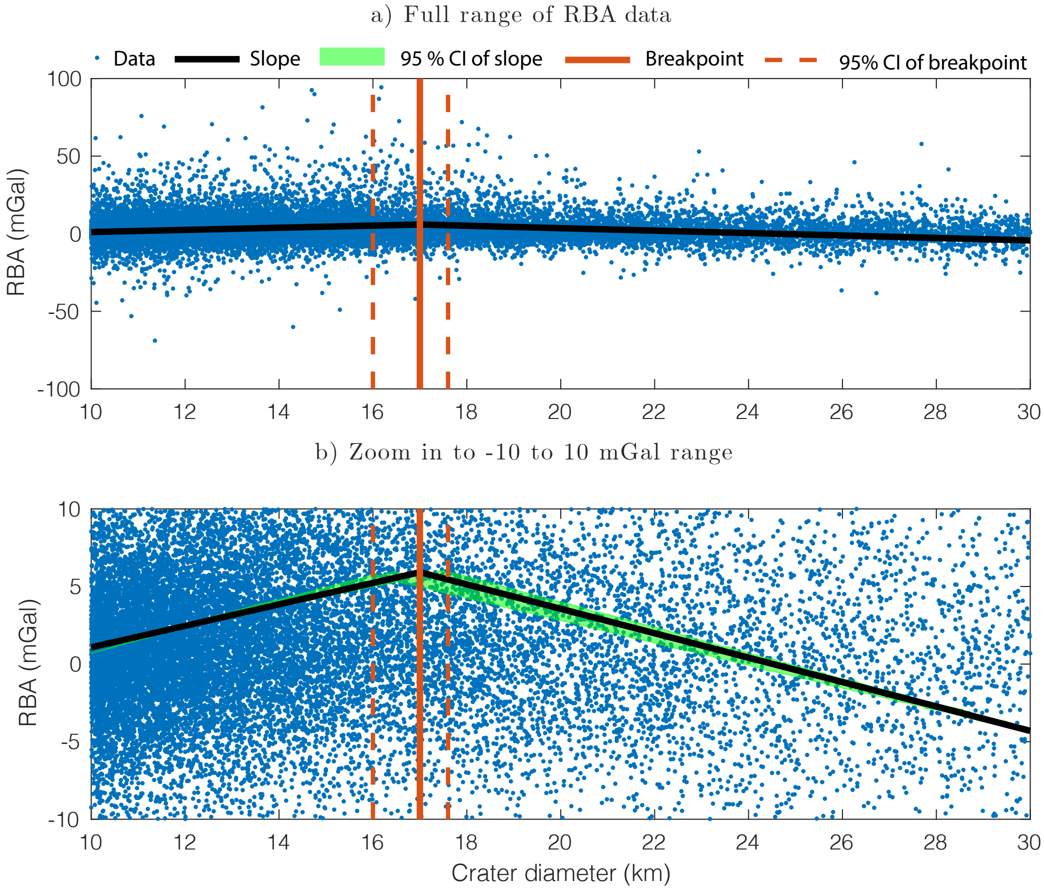

Figure S7. RBA data obtained using a value of 2550 kg/m^3 as the mean density of the lunar crust and truncation degree 640 without tapering. The breakpoint is located at $D = 17^{+0.6}_{-1.0}$ km.

Figure S8 shows the RBA data obtained using a truncation degree 660 and no cosine taper. The location of the break in slope of the best fit model is $D = 16.4^{+1.6}_{-0.2}$ km showing

that only the confidence interval changed, compared to the break in slope obtained using truncation degree 650 and no taper, $D = 16.4^{+1.2}_{-0.8}$ km.

In general, the use of a cosine taper does not have an effect on the location of the break in slope while the truncation degree might have a minor effect. Because the effect on the location of the breakpoint is small, the proposed porosity boundary depth at 3 to 5 km is robust to the use of a cosine taper and truncation degrees tested.

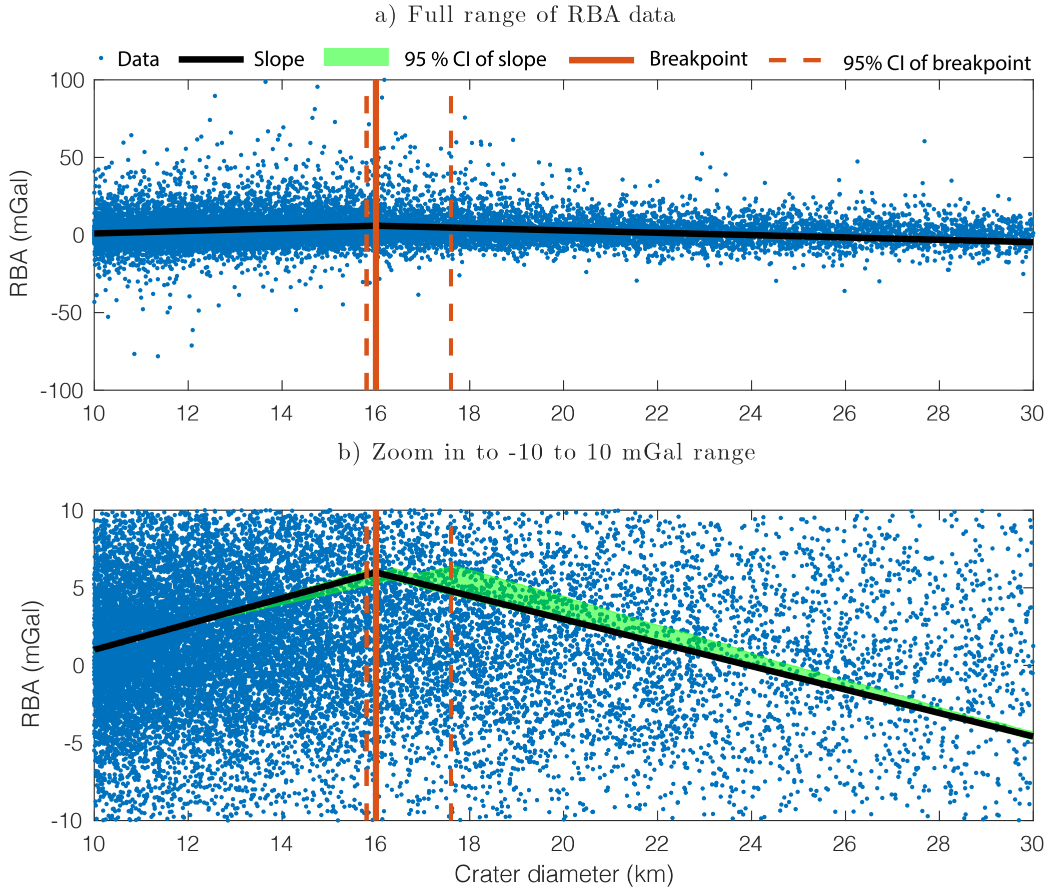

Figure S8. RBA data obtained using a value of 2550 kg/m^3 as the mean density of the lunar crust and truncation degree 660 without tapering. The breakpoint is located at $D = 16.4^{+1.6}_{-0.2}$ km.

References

Goossens, S., Sabaka, T. J., Wieczorek, M. A., Neumann, G. A., Mazarico, E., Lemoine, F. G., ... Zuber, M. T. (2020). High-Resolution Gravity Field Models from GRAIL Data and Implications for Models of the Density Structure of the Moon's Crust. *Journal of Geophysical Research: Planets*, *125*(2), 1–31. Retrieved from https://doi.org/10.1029/2019JE006086 doi: 10.1029/2019JE006086

Head, J. W. I., Fassett, C. I., Kadish, S. J., Smith, D. E., Zuber, M. T., Neumann, G. A., & Mazarico, E. (2010). Global Distribution of Large Lunar Craters : Implications for Resurfacing and Impactor Populations. *Science*, *329*(5998), 1504–1508. Retrieved from https://doi.org/10.1126/science.1195050 doi: 10.1126/science.1195050

Konopliv, A. S., Park, R. S., Yuan, D.-N., Asmar, S. W., Watkins, M. M., Williams, J. G., ... Zuber, M. T. (2014). High-resolution lunar gravity fields from the GRAIL Primary and Extended Missions. *Geophysical Research Letters*, *41*(5), 1452–1458. Retrieved from http://doi.wiley.com/10.1002/2013GL059066 doi: 10.1002/2013GL059066.Received

Main, I. G., Leonard, T., Papasouliotis, O., Hatton, C. G., & Meredith, P. G. (1999). One slope or two? Detecting statistically significant breaks of slope in geophysical data, with application to fracture scaling relationships. *Geophysical Research Letters*, *26*(18), 2801–2804. Retrieved from https://agupubs.onlinelibrary.wiley.com/doi/abs/10.1029/1999GL005372 doi: 10.1029/1999GL005372

Robbins, S. J. (2019). A New Global Database of Lunar Impact Craters >12km: 1. Crater Locations and Sizes, Comparisons With Published Databases, and Global

Analysis. *Journal of Geophysical Research: Planets*, *124*(4), 871–892. Retrieved from https://doi.org/10.1029/2018JE005592 doi: 10.1029/2018JE005592

Robert, C. P., Chopin, N., & Rousseau, J. (2009). Harold Jeffreys's theory of probability revisited. *Statistical Science*, *24*(2), 141–172. doi: 10.1214/09-STS284

Soderblom, J. M., Evans, A. J., Johnson, B. C., Melosh, H. J., Miljkovi, K., Phillips, R. J., ... Zuber, M. T. (2015). The fractured Moon: Production and saturation of porosity in the lunar highlands from impact cratering. *Geophysical Research Letters*, *42*(17), 6939–6944. Retrieved from https://agupubs.onlinelibrary.wiley.com/doi/full/10.1002/2015GL065022 doi: 10.1002/2015GL065022

Wieczorek, M. A., Neumann, G. A., Nimmo, F., Kiefer, W. S., Taylor, G. J., Melosh, J., ... Zuber, M. T. (2013). The Crust of the Moon as Seen by GRAIL. *Science*, *339*(February), 671–676. doi: 10.1126/science.1231530
